# Removal of the N-Glycosylation Sequon at Position N116 Located in p27 of the Respiratory Syncytial Virus Fusion Protein Elicits Enhanced Antibody Responses after DNA Immunization

**DOI:** 10.3390/v10080426

**Published:** 2018-08-14

**Authors:** Annelies Leemans, Marlies Boeren, Winke Van der Gucht, Isabel Pintelon, Kenny Roose, Bert Schepens, Xavier Saelens, Dalan Bailey, Wim Martinet, Guy Caljon, Louis Maes, Paul Cos, Peter Delputte

**Affiliations:** 1 Laboratory of Microbiology, Parasitology and Hygiene, University of Antwerp, B-2610 Antwerp, Belgium; annelies.leemans@uantwerpen.be (A.L.); marlies.boeren@uantwerpen.be (M.B.); winke.vandergucht@uantwerpen.be (W.V.d.G.); guy.caljon@uantwerpen.be (G.C.); louis.maes@uantwerpen.be (L.M.); paul.cos@uantwerpen.be (P.C.); 2 Laboratory of Cell Biology and Histology, University of Antwerp, B-2610 Antwerp, Belgium; isabel.pintelon@uantwerpen.be; 3 Medical Biotechnology Centre, VIB, B-9052 Ghent, Belgium; kenny.roose@vib-ugent.be (K.R.); bert.schepens@vib-ugent.be (B.S.); xavier.saelens@vib-ugent.be (X.S.); 4 Department of Biomedical Molecular Biology, Ghent University, B-9052 Ghent, Belgium; 5 The Pirbright Institute, Surrey GU24 0NF, UK; dalan.bailey@pirbright.ac.uk; 6 Laboratory of Physiopharmacology, University of Antwerp, B-2610 Antwerp, Belgium; wim.martinet@uantwerpen.be

**Keywords:** class I fusion protein, virus glycosylation, DNA immunization, humoral responses, pneumovirus, vaccine

## Abstract

Prevention of severe lower respiratory tract infections in infants caused by the human respiratory syncytial virus (hRSV) remains a major public health priority. Currently, the major focus of vaccine development relies on the RSV fusion (F) protein since it is the main target protein for neutralizing antibodies induced by natural infection. The protein conserves 5 N-glycosylation sites, two of which are located in the F2 subunit (N27 and N70), one in the F1 subunit (N500) and two in the p27 peptide (N116 and N126). To study the influence of the loss of one or more N-glycosylation sites on RSV F immunogenicity, BALB/c mice were immunized with plasmids encoding RSV F glycomutants. In comparison with F WT DNA immunized mice, higher neutralizing titres were observed following immunization with F N116Q. Moreover, RSV A2-K-line19F challenge of mice that had been immunized with mutant F N116Q DNA was associated with lower RSV RNA levels compared with those in challenged WT F DNA immunized animals. Since p27 is assumed to be post-translationally released after cleavage and thus not present on the mature RSV F protein, it remains to be elucidated how deletion of this glycan can contribute to enhanced antibody responses and protection upon challenge. These findings provide new insights to improve the immunogenicity of RSV F in potential vaccine candidates.

## 1. Introduction

Worldwide, the human respiratory syncytial virus (hRSV) is the most common cause of lower respiratory tract disease in infants and young children [1,2]. RSV has an estimated incidence of 33.8 million infections annually in children younger than 5 years [3]. An estimated 3.4 million children are hospitalized and up to 200,000 cases are fatal [3]. RSV belongs to the genus *Orthopneumovirus* in the family Pneumoviridae and has a non-segmented negative-stranded RNA genome [4]. This genome codes for 11 proteins, three of which are displayed on the viral envelope: the G glycoprotein, the fusion (F) protein and the small hydrophobic (SH) protein [1]. RSV F shows 89% homology among strains of different subtypes and is thereby the most conserved RSV envelope protein [5]. Moreover, the F protein is essential for RSV entry by mediating fusion between virion and target cell membranes [6]. RSV neutralization by human serum is predominantly obtained by the activity of RSV F-specific antibodies [7] and monoclonal antibodies (mAbs) specific for the RSV F protein (palivizumab) can reduce hospitalization due to severe bronchiolitis and pneumonia when administered prophylactically to high-risk infants [8,9]. As such, vaccine research is mainly focused on the RSV F protein. However, to date no hRSV vaccine is available yet and treatment is mainly supportive by maintenance of hydration and oxygenation [10]. 

RSV F is a type I glycoprotein that is initially synthesized as an inactive precursor (F0) and is post-translationally cleaved by furin-like proteases into F1 (50 kDA) and F2 (20 kDA) subunits, which are covalently linked by two disulphide bridges to form the mature and biologically active form of this glycoprotein [11,12]. Like many viral envelope proteins, RSV F is co- and post-translationally modified by the addition of N-linked glycans during its transport through the secretory pathway [13]. Five potential N-glycosylation sites, with the consensus sequence N-X-S/T [14], are conserved among the F proteins of different RSV isolates, of which two sites (N27 and N70) are located in the F2 subunit and only one site (N500) is located in the F1 subunit [15]. Two additional sites N116 and N126 are located in the small peptide p27 which is positioned between F1 and F2 in the precursor F0 protein and released from the mature protein by proteolytic cleavage [16]. Depending on the strain, an additional potential N-glycosylation site is found at positon N120 within p27.

N-linked glycosylation is an important post-translational modification of glycoproteins that is involved in different processes which determines their structure and activity [17]. Likewise, N-glycosylation of viral spike proteins is a prerequisite for proper folding and subsequent trafficking of the protein [18]. For most viral glycoproteins their stability is assured by glycosylation; either by an individual N-glycan or multiple N-glycan structures [19]. Moreover, translocation to the cell surface and interaction with host cells including receptor binding and fusion can be influenced by N-linked glycans [18,20,21,22]. For example, for RSV, the glycan structure positioned at N500 at the RSV F protein was shown to be important for the fusion activity, since reduced fusion activity was observed after removal of the corresponding sequon [15,23]. Additionally, the immunogenicity of viral glycoproteins is often determined by their glycosylation profile. The attachment of N-linked glycans can affect the recognition of antibodies by shielding antigenic sites on the protein [24,25,26,27,28,29]. For example, N-linked glycans might shield up to 52% of the RSV F protein surface for antibody recognition [30]. Removal of specific N-linked glycans on viral glycoproteins can elicit more potent neutralizing antibody responses and may be an interesting approach for vaccine design. In the context of DNA vaccines or live-attenuated vaccines (LAVs), this approach was already broadly investigated for multiple viruses [25,27,29,31,32]. Interestingly, mice immunized with a specific bRSV F glycomutant showed higher antibody responses compared with the wild type (WT) bRSV F protein [24]. However, it is not clear how the individual N-linked glycans impact the immunogenicity of the hRSV fusion protein and to which extent the observations of the glycomutant bRSV F protein immunizations can be extrapolated to hRSV F. 

In this report, the effects of loss of conserved RSV F N-glycosylation sites on cell surface expression, fusiogenicity and antigenicity were analysed. In addition, we studied the immunogenicity of the RSV F N-glycomutants by intramuscular immunization of mice with plasmids encoding glycomutant RSV F proteins. Subsequent challenge of the immunized mice with RSV A2-line19F was performed to compare the protective effect of the induced immune responses. 

## 2. Materials and Methods

### 2.1. Cells, Virus and Antibodies

BSR T7/5 cells were a gift from K.K Conzelmann (Max-von-Pettenhofer-Institut, Munich, Germany) and were grown in Glasgow’s minimal essential medium (GMEM), supplemented with 10% heath-inactivated foetal bovine serum (iFBS) and 2% minimal essential amino acids (Thermo Fisher Scientific, Waltham, MA, USA). The HEK293T cell line was obtained from the ATCC. The cells were grown in DMEM supplemented with 10% iFBS. The RSV reference strain A2 was obtained from the Biodefense and Emerging Infections Research Resources Repository (BEI resources, Manassas, VA, USA) and propagated in a human epithelial cell line (HEp-2), obtained from ATCC and grown in Dulbecco’s modified Eagle medium (DMEM) supplemented in 10% iFBS (Thermo Fisher Scientific). RSV cDNA-containing BAC pSynkRSV-line19F was obtained from M.L. Moore (Emory University School of Medicine, Atlanta, GA, USA) and recovered as described before to obtain strain RSV A2-K-line19F [33]. As RSV polyclonal antibodies, goat anti-RSV (Virostat, Westbrook, ME, USA) and RSV reference antiserum (BEI resources) were used. Palivizumab leftovers were provided by the Department Paediatrics of the Antwerp University Hospital (S. Verhulst). RSV F-specific mAbs AM14, 101F, D25 and MPE8 were provided by J.A. Melero (Centro Nacional de Microbiología and CIBER de Enfermedades Respiratorias, Instituto de Salud Carlos III, Majadahonda, Madrid, Spain), J.S. McLellan (Department of Biochemistry, Geisel School of Medicine at Dartmouth, Hanover, NH, USA) and B.S. Graham (Vaccine Research Centre, National Institute of Allergy and Infectious Diseases, National Institutes of Health, Bethesda, MD, USA). Corresponding secondary antibodies were obtained from Thermo Fisher Scientific and include Alexa Fluor (AF) 555 donkey anti-goat IgG, AF488 and AF647 goat anti-human IgG, AF647 goat anti-mouse IgG and HRP-conjugated goat anti-mouse and goat anti-human IgG.

### 2.2. Construction and Expression of Recombinant RSV F Proteins

The RSV F glycosylation mutants were obtained by changing the asparagine (N) codons (AAT/AAC) at position N27, N70, N116, N126 or N500 into a glutamine (Q) codon (CAA/CAG). A triple and penta mutant F protein was developed by replacing 3 asparagine codons (N27/70/500) and all 5 conserved N codons into Q codons (N27/70/116/126/500), respectively. Synthesis of the recombinant codon-optimized RSV line19F proteins was performed by Genscript and delivered in pUC57 simple, a commonly used plasmid for cloning. Appropriate restriction enzymes (New England Biolabs, Ipswich, MA, USA) were used to excise the DNA from the vector and subsequently the DNA was ligated into a mammalian expression vector pBUDCE4.1 (Thermo Fisher Scientific) or pCAXL by using T4 DNA ligase (New England Biolabs). The sequences of the recombinant RSV F proteins were confirmed by DNA sequencing (VIB Genetic Service Facility, University of Antwerp). The resulting plasmids were transfected in BSR T7/5 cells by using ViaFect^TM^ Transfection Reagent (Promega, Madison, WI, USA) to obtain expression of the recombinant RSV F proteins. Briefly, BSR T7/5 cells were seeded to be approximately 75% confluent at the time of transfection. A 3:1 ratio transfection reagent:plasmid DNA was used and diluted in Opti-MEM (Thermo Fisher Scientific). After a 20-min incubation period, the transfection complexes were added to the cells in complete GMEM and further incubated at 37 °C.

### 2.3. Western Blot Analysis

BSR T7/5 cells were seeded in 6-well plates to reach a confluency of approximately 75% at the time of transfection. Transfection of plasmid DNA with ViaFect^TM^ Transfection Reagent (Promega) was performed as described above. After an incubation period of 24 h the cells were washed with ice-cold PBS following lysis with RIPA buffer (Millipore, Burlington, MA, USA) and protease inhibitors (Roche, Basel, Switzerland). Cells were scraped and incubated at 4 °C for 30 min and centrifuged (13,000× *g*, 10 min, 4 °C). Lysates were mixed 1:1 with Laemmli sample buffer (Bio-Rad, Hercules, CA, USA). Reducing agent β-mercaptoethanol was added, for analysis under reducing conditions. Samples were treated with PNGase F (New England Biolabs, Ipswich, MA, USA) according to the manufacturer’s instructions. After boiling the mixtures, the cell lysates were loaded and separated on 4–20% or any kD polyacrylamide gels (Bio-Rad) and transferred to a Immobilon-P transfer membrane (Millipore). RSV F proteins were visualized with palivizumab and HRP-conjugated goat anti-human IgG. Monoclonal anti β-actin antibody (Sigma, Tokyo, Japan) to detect β-actin levels served as loading control. Chemiluminescence was measured after incubation with a chemiluminescent substrate (Thermo Fisher Scientific) using a GenoPlex Chemi camera (VWR, Radnor, PA, USA).

### 2.4. Immunofluorescence Analysis of Surface Expression

Cells were co-transfected with plasmids expressing WT or glycomutant RSV F proteins and a GFP-expressing plasmid EGFP-N3 (Clontech, Mountain View, CA, USA) as transfection control. Transfected cells on coverslips in 24-well plates were incubated with polyclonal goat serum for 1 h at 4 °C. Afterwards, the cells were fixed with 4% paraformaldehyde (PF) and permeabilized with 0.5% Triton X-100. Donkey anti-goat IgG conjugated with AF555 was used to visualize surface-expressed antigen-antibody complexes by confocal microscopy (Leica SP8, Bethesda, MD, USA).

### 2.5. Flow Cytometric Analysis

BSR T7/5 cells were seeded in 6-well plates to be subconfluent after overnight incubation at 37 °C and subsequently co-transfected with plasmids expressing WT or glycomutant RSV F proteins and a GFP-expressing plasmid EGFP-N3 (Clontech) as described above. After 24 h, transfected cells were resuspended in ice-cold PBS and pelleted by centrifugation (210× *g*, 10 min, 4 °C). The pellet was washed once with PBS and then incubated with human anti-RSV reference serum (BEI resources) or the RSV F-specific monoclonal antibodies palivizumab, D25, MPE8, AM14 and 101F at a concentration of 5 µg/mL for 1 h at 4 °C. To remove unbound antibodies the cells were washed two times with PBS. Then, goat anti-human or goat anti-mouse AF647-conjugated secondary antibodies were added to the cell pellets for 1 h at 4 °C, washed with PBS and analysed by flow cytometry with a FACSCalibur. Forward-scattered light (FSC), side-scattered light, the green fluorescence (FL-1) and far-red fluorescence signal (FL-4) were stored for further analysis. Mean fluorescence intensity (MFI) of the mutant RSV F transfected cells was calculated and presented as values relative to the MFI of the WT RSV F protein (100%) or presented as absolute values. 

### 2.6. Fusion Assay

Transfection of BSR T7/5 cells was performed as described above. After 24 h incubation, the cells were analysed by fluorescence microscopy after immunofluorescence staining of the cells with palivizumab and AF488-conjugated goat anti-human IgG. Staining of the cells with DAPI was performed to count the syncytia (cells with more than two nuclei) of 100 transfected cells.

HEK293T cells were plated out at a cell density of 7.5 × 10^5^ cells per well in 6-well plates. The following day, ‘effector’ cells were transfected with 2 μg of WT or glycomutant RSV F plasmids, as well as 250 ng of the 1–7 fragment of rLuc-GFP [34]. In separate plates, ‘target’ cells were transfected with 250 ng of the 8–11 fragment of rLuc-GFP. All transfections were executed using the TransitX transfection reagent (Mirus, Madison, WI, USA) as per the manufacturer’s protocol for DNA plasmid transfection. Following 48 h of incubation (37 °C, 5% CO_2_), ‘RSV effector’ and ‘target’ cell populations were separately washed, resuspended in media, counted and then co-cultured at a ratio of 1:1 in white-walled 96-well plates to a final density of 1 × 10^5^ cells per well. Sixteen–twenty-four hours later, the activity of the reconstituted Renilla luciferase activity in fused cells was measured (using a Promega GloMax multi-mode plate reader) by removing the media and adding 2 μg/mL of cell-permeable coelenterazine 400A (Biotium, Fremont, CA, USA) in PBS to a final volume of 100 μL. Of note, control wells contained the same combination of effector and target cells; however, the effector cells in this instance were not transfected with RSV F expression constructs. Five co-culturing replicates were performed for each biological condition.

### 2.7. Immunization of Mice with Plasmid DNA and RSV Challenge

Female 7–8 weeks old BALB/c mice (Janvier, France) were randomly allocated to 10 individually ventilated cages of 6 animals each. Food (Carfil, Belgium) and drinking water were available ad libitum. Mice were anesthetized with 5% isoflurane (Halocarbon^®^, Atlanta, GA, USA) before they were immunized intramuscularly in both quadriceps muscle with 100 µg of Endotoxin-free plasmid DNA in total (dissolved in 100 µL 0.9% NaCl solution) at day 0 and day 21. The 10 different treatment groups included pCAXL RSV F WT, N27Q, N70Q, N116Q, N126Q, N500Q, N27/70/116/126/500Q and as negative control the empty pCAXL vector. Mice were anesthetized with 5% isoflurane before blood was collected via retro-orbital bleeding at day 0, day 21, day 35 and day 56. The blood was left to clot in a serum clot activator tube (Sarstedt, Nümbrecht, Germany) at room temperature for 30 min and supernatant was collected after centrifugation (5 min, 10,000× *g*). 

RSV challenge was performed by intranasal inoculation of 10^6^ PFU RSV A2-K-line19 diluted in 100 µL HBSS. Five days post challenge (day 61), serum was collected before mice were sacrificed by CO_2_ and lungs were removed. The left lung was homogenized in HBSS for the determination of RSV RNA levels (see below) while the right lung was fixed with formaldehyde for the preparation of paraffin slides (see below). The animal studies were approved by the Animal Ethical Committee of the University of Antwerp (UA-ECD 2015-63; 1 October 2015).

### 2.8. Antibody Responses and Neutralization Assay

RSV-infected HEp-2 cells (MOI = 0.5) were used as antigen and propagated in 96-well microtiter plates (Falcon, Corning, NY, USA,). After methanol fixation, permeabilization with 0.5% Triton X-100 and blocking with 1% bovine serum albumin (BSA) (Santa Cruz Technologies, Santa Cruz, CA, USA), two-fold dilutions (starting from 1:10) of the heat-inactivated mice serum were added to the cells and incubated for 1 h at 37 °C. Afterwards, the cells were stained with HRP-conjugated goat anti-mouse IgG. 3,3′diaminobenzidine (DAB) (Sigma) was added to the cells as a substrate for HRP. Light microscopic analysis was performed to determine the antibody titres of the serum and are displayed as log 2 of the lowest concentration were staining of RSV-infected cells was observed.

Plaque reduction neutralization tests (PRNT) were performed to determine the neutralizing antibody titres. Prior to inoculation of subconfluent HEp-2 monolayers, 2-fold dilutions of heat-inactivated serum in duplicate were incubated with virus inoculum for 1 h at 37 °C. Binding of the virus was allowed for 2 h at 37 °C and afterwards an overlay of DMEM + 0.6% Avicel (FMC Biopolymer, Philadelphia, PA, USA) was added to the cells. After 3 days incubation at 37 °C, cells were fixed with 4% PF, permeabilized with Triton X-100 and blocked with 1% BSA. Plaques were stained with palivizumab and HRP-conjugated goat anti-human IgG. Chloronapthol (Thermo Fisher Scientific) was used to visualize the plaques. Neutralization titres were calculated by the concentration resulting in a 50% reduction compared to control wells.

### 2.9. Determination of Lung Viral Titre by qRT-PCR

To determine the lung RSV load by qRT-PCR, left lungs were excised and total RNA from the cleared lung homogenates was prepared by using the High Pure RNA tissue Kit (Roche) according to the manufacturer’s instructions. Next, cDNA was prepared by the use of random hexamer primers and the Transcriptor First strand cDNA synthesis kit (Roche). The relative levels of genomic RSV M cDNA were determined by qRT-PCR using primers specific for the RSV A2 M gene (5′TCACGAAGGCTCCACATACA3′ and 5′GCAGGGTCATCGTCTTTTTC3′) and a nucleotide probe (#150 Universal Probe Library, Roche) labelled with fluorescein (FAM) at the 5′-end and with a dark quencher dye near the 3′-end. 

### 2.10. Immunohistochemistry

Right lungs were excised, fixed in 4% formaldehyde (pH 7.4) and paraffin embedded. Sections of 5 µM thickness were stained with goat anti-RSV IgG (Virostat) and a Vectastain ABC kit (Vector Laboratories, Burlingame, CA, USA) was used to detect RSV antigens in the lung.

### 2.11. Statistical Analysis

Data are presented as means (±SD) of the indicated independent repeats. To determine the significance between the values of the WT group and one of the different mutant groups, data were analysed pairwise with a student’s *t*-test using GraphPad Prism 6 (La Jolla, CA, USA). The interaction between the recombinant proteins and the RSV F-specific mAbs was analysed by a two-way ANOVA using Genstat v18 [35].

## 3. Results

### 3.1. Expression Analysis of the RSV F Glycomutants and the Effect of N-Sequon Removal on RSV F-Specific mAb Recognition

Deletion of a specific N-glycosylation site of the RSV F protein was obtained by the substitution of the asparagine (N) codon into a glutamine (Q) codon. The side chains of Q are very similar to N and therefore Q is a good substitute. All 5 conserved N-glycosylation sites were individually converted from N-X-S/T into Q-X-S/T resulting in the following 5 RSV F constructs (F N27Q, F N70Q, F N116Q, F N126Q, F N500Q). Additionally, plasmids encoding a mutant F sequence lacking 3 (F N27-70-500Q) or all 5 conserved N-glycosylation sites (F N27-70-116-126-500Q) were generated. After subcloning the sequences into a mammalian expression plasmid pCAXL and DNA sequence verification, the plasmids were transiently transfected into BSR T7/5 cells and RSV F expression was monitored. Indirect immunofluorescence staining showed that all RSV F constructs were expressed in BSR T7/5 cells.

**Figure 1:**
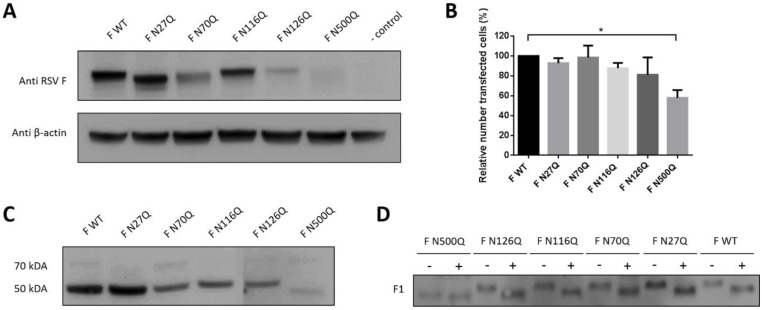
Expression analysis of recombinant respiratory syncytial virus (RSV) F proteins. BSR T7/5 cells were transfected with pCAXL encoding RSV F glycosylation mutants. Cells were lysed and proteins were separated by SDS page or cells were analysed by immunofluorescence staining. (**A**) Proteins were separated under non-reducing conditions and Western blot was developed with palivizumab and anti-actin IgG. (**B**) Cells were stained with palivizumab and AF488-conjugated goat anti-human IgG to determine the relative number of transfected cells (%). Data represents the mean (±SD) of three independent repeats * *p* < 0.05 (Student’s unpaired two-tailed *t* test). (**C**,**D**) Cell lysates were separated under reduced conditions in panel C and treated with PNGase F (+) or left untreated (−) for 2 h at 37 °C in panel D. Western blots were probed with palivizumab.

Depending on the extent of the glycan, protein deglycosylation can reduce their molecular weight, on average 2 kDA for a complex N-glycan in mammalian cells. Gel electrophoresis of lysed transfected cells under non-reduced conditions was performed to separate the glycomutants by molecular weight. WT and all 5 single N-glycan mutants could be visualized with palivizumab in Western blot as single bands with an apparent molecular weight of approximately ±70 kDA (Figure 1A). The electrophoretic mobility of mutants F N27Q and F N500Q was clearly increased compared to WT F, whereas a change in apparent molecular weight was less clear for the mutant F N70Q, suggesting variable sizes or occupancy of N-glycans at the different positions. Deletion of N-glycan sequons at positions N116 and N126 did not result in a detectable molecular weight change on Western blot compared to WT F. Lower expression levels were obtained for mutants F N70Q, F N126Q and F N500Q. Lower levels could be due to a reduced number of transfected cells, that is, observed after mutation of N-glycosylation site N500 (Figure 1B). Similar results were obtained in transfected human HEK-293T cells (Figure S2). Gel electrophoresis under reducing conditions showed for each mutant efficient processing to the mature F protein, associated with cleavage of the F0 and detection of the single F1 band of 50 kDA (Figure 1C). Due to the removal of the glycan at position N500 at the F1 subunit, a lower molecular weight was observed for the F N500Q mutant. Treatment with PNGase F resulted in a reduced electrophoretic mobility of F1 for WT F and all mutants, due to the removal of N-glycan structures by this enzyme (Figure 1D).

**Figure 2:**
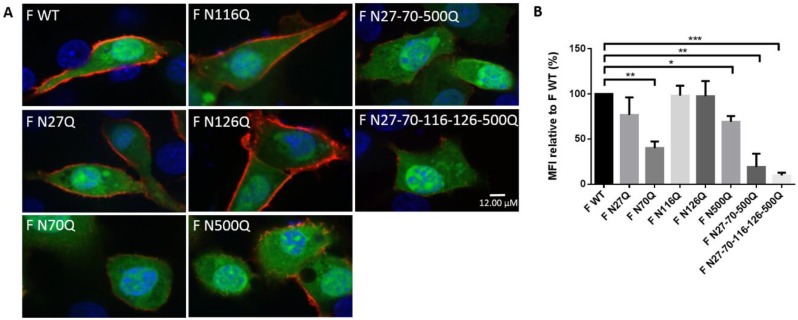
Surface expression analysis of RSV F glycosylation mutants. (**A**) Immunofluorescence staining of cell surface expression of glycomutant RSV F proteins. BSR T7/5 cells were transfected with a pCAXL plasmid encoding RSV F glycosylation mutants and a GFP-expressing plasmid (EGFP-N3) (green). Twenty-four h after transfection, cells were fixed and surface-expressed RSV F proteins were visualized by polyclonal goat anti-RSV antibodies and secondary donkey anti-goat IgG (AF555) (red). The nuclei were stained with DAPI (blue). Images were acquired by confocal fluorescence microscopy, scale bar = 12 μM (**B**) Flow cytometric analysis of BSR T7/5 cells expression glycosylation mutants of the RSV F protein. BSR T7/5 cells were transfected with a pCAXL plasmid encoding WT or one of the glycosylation mutants of the RSV F protein and a GFP-expressing plasmid (EGFP-N3). Twenty-four h after transfection, the cells were detached and stained with human RSV antiserum at 4 °C to assure surface staining only. Secondary anti-human IgG conjugated with AF647 was used to detect RSV F proteins. Fluorescence intensities of fluorescence channel (FL) 4 were measured of 5000 cells expressing GFP (FL1). Surface expression is expressed as the MFI relative to F WT expression. Data represents the means (±SD) from 3 independent repeats. * *p* < 0.05; ** *p* < 0.01; *** *p* < 0.001 (Student’s unpaired two-tailed *t* test).

To evaluate the impact of deglycosylation on RSV F surface expression, fluorescence intensities of transfected BSR T7/5 were measured after staining surface RSV F proteins with goat anti-RSV serum. Cells were co-transfected with a GFP-expressing plasmid which served as a marker of transfected cells and as a control for quantification of the number of transfected cells, to exclude the effect of variable transfection efficiencies of the F constructs. Confirmation by immunofluorescence staining was performed and co-expression was observed for the majority of the transfected cells. In addition, intracellular staining of the glycomutant RSV F proteins ruled out the influence of a reduced binding capacity due to deglycosylation. Representative images of immunofluorescence cell surface staining are shown in Figure 2A. Flow cytometry analysis showed surface expression of all mutant RSV F proteins. However, significant reductions were observed for mutants F N70Q, F N500Q, F N27-70-500Q and F N27-70-116-126-500Q (Figure 2B). The latter two in particular showed low surface intensities compared to the parental protein.

**Figure 3:**
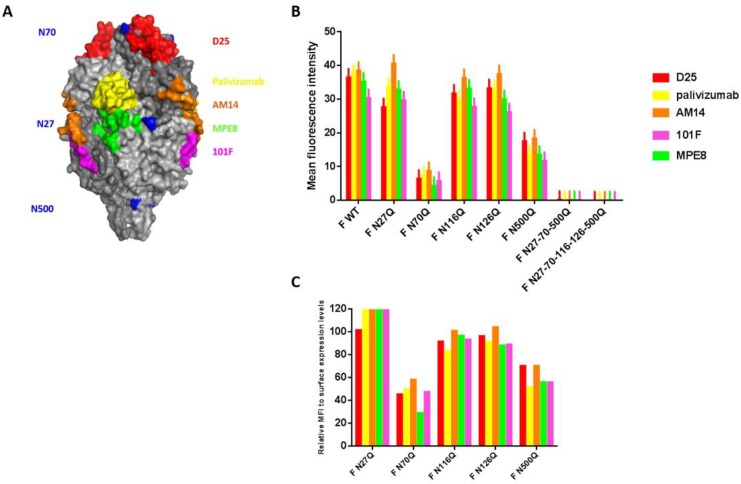
Evaluation of the effect of loss of N-glycosylation sequons on the recognition of RSV F-specific mAbs. (**A**) Surface representation of prefusion F. N-glycan structures are indicated in blue. MAb epitopes are highlighted in different colours. D25, red; palivizumab, yellow; AM14, orange; MPE8, green; 101F, magenta. (**B**) Flow cytometric analysis of BSR T7/5 cells expression glycosylation mutants of the RSV F protein. BSR T7/5 cells were transfected with a pCAXL plasmid encoding one of the glycosylation mutants of the RSV F protein and a GFP-expressing plasmid (EGFP-N3). After 24 h incubation the cells were detached and stained with RSV F-specific mAbs (palivizumab, AM14, D25, MPE8 or 101F) at 4 °C. Secondary anti-human or anti-mouse IgG conjugated with AF647 were used to detect RSV F proteins. Fluorescence intensities of fluorescence channel (FL) 4 were measured of 5000 cells expressing GFP (FL1). The graph shows the surface expression expressed as the MFI (±SEM) from 3 independent repeats. There was no significant interaction between the recombinant F proteins and the binding of the different mAbs (*p* = 0.741; F-test; two-way ANOVA). (**C**) Normalization towards protein surface expression levels expressed as relative MFI values to surface expression levels after staining with human RSV antiserum.

Additionally, the influence of RSV F glycosylation on the recognition by well-described RSV F-specific mAbs that target different antigenic sites on the RSV F protein was studied (Figure 3A) [36,37,38,39,40]. Deletion of N-glycans could affect recognition of the neutralizing epitopes. The co-transfected cells were stained with the mAbs and further analysed by flow cytometry (Figure 3B). For F WT and each mutant F protein, no significant differences in recognition were observed between the different RSV F-specific mAbs. Moreover, reduction in surface staining observed with the mAb immune-staining of transfected cells correlated with the reduction observed after staining with polyclonal human serum of Figure 1 for mutants F N27Q, F N116Q and F N126Q (Figure 3C). This suggests that the reduced staining with the mAbs is because of lower cell surface expression of the mutant F proteins and not because of the removal of the RSV F N-glycosylation sequons affects the accessibility of the epitopes that correspond to the mAbs Palivizumab, AM14, D25, MPE8, 101F. Reduced detection of mutants F N70Q and F N500Q with the mAbs are also in part a consequence of reduction in surface expression, however, normalization against the staining with the pAb also showed reductions in recognition of all different mAbs. 

### 3.2. RSV F Fusion Activity Is Determined by the N-Glycan at Position N500

**Figure 4:**
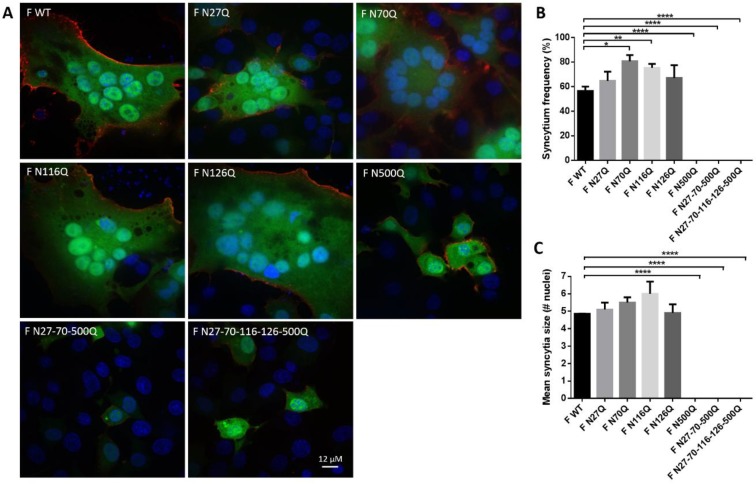
The effect of loss of RSV F N-glycosylation sites on syncytia formation. After 24 h incubation of BSR T7/5 cells transfected with glycomutant RSV F plasmids, the cells were fixed and permeabilized. RSV F proteins were stained with palivizumab and secondary goat anti-human IgG AF488. Syncytia were visualized by staining the nuclei with DAPI and further analysed by fluorescence microscopy. (**A**) Representative confocal images of the syncytia are shown for each glycomutant RSV F protein, scale bar = 12 µM. (**B**) Syncytia frequency and (**C**) syncytium size of 100 transfected cells was counted. Data represents the mean (±SD) of three independent repeats. * *p* < 0.05; ** *p* < 0.01; **** *p* < 0.0001 (Student’s unpaired two-tailed *t* test).

Previous observations about the role of glycosylation in the fusion capacity of the RSV F protein were confirmed in our study by two different assays [15,23]. Twenty-four h after transfection of BSR T7/5 cells, syncytia formation could be visualized by immunofluorescence staining of the RSV F protein combined with staining of the nuclei with DAPI (Figure 4A). Fused cells were considered a syncytium when containing more than 2 nuclei. Syncytium formation was enhanced for mutant constructs F N70Q and F N116Q (Figure 4B). For all constructs, large syncytia with a mean of 5 nuclei or more were detectable, except for cells transfected with RSV F plasmids containing mutation N500Q, either the single mutant as well as the triple and the pentamutant (Figure 4C). Either for the single F N500Q mutant as well as the triple mutant and the pentamutant, no cells with more than two nuclei were observed. 

**Figure 5:**
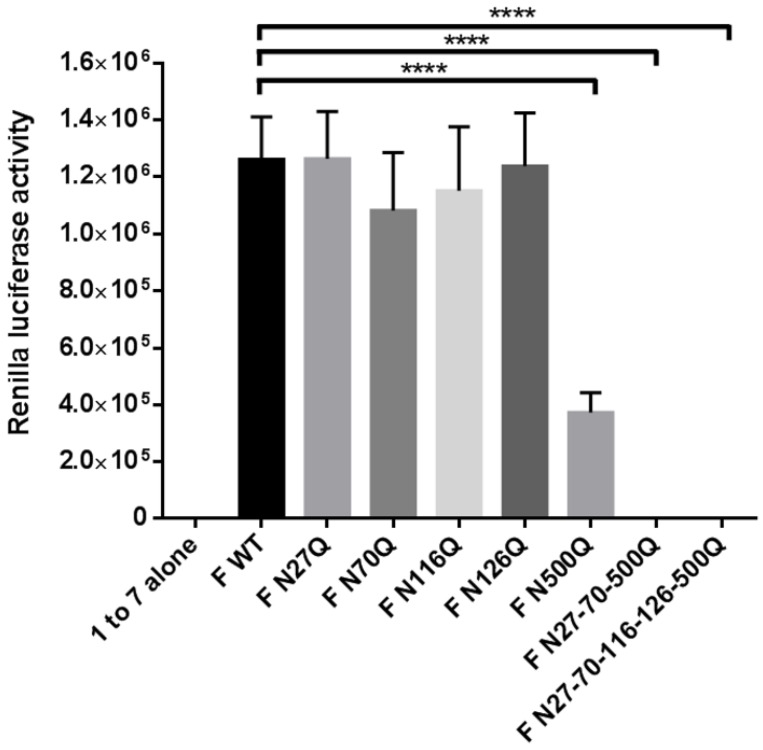
Dual split protein assay to measure RSV F fusion activity. HEK293T cells were transfected with one of the RSV F plasmids as well as the 1–7 fragment of rLuc-GFP or with the 8–11 fragment of rLuc-GFP which corresponds with effector cells and target cells, respectively. After 48 h incubation, the effector and target cells were washed, resuspended in media and further co-cultured. The activity of the reconstituted Renilla luciferase in fused cells was measured after 16–24 h by adding cell-permeable coelenterazine. Five co-culturing replicates were performed for each biological condition. Data represents the mean values (±SD) of five co-culturing replicates. **** *p* < 0.0001 (Student’s unpaired two-tailed *t* test).

Secondly, a dual split protein assay was performed to measure the fusion capacity of the recombinant RSV F proteins compared with the WT F proteins. This assay is highly sensitive and was already widely used to study the impact of specific mutations of viral glycoproteins on their fusion activity [41,42,43]. Cells transfected with the RSV F plasmids and the 1–7 fragment of rLuc-GFP were co-cultured with target cells transfected with the 8–11 fragment of rLuc-GFP to initiate fusion. The fusion activity was expressed as the activity of the reconstituted Renilla luciferase (Figure 5). Only the mutants lacking the N500 glycan sequon showed very low or negligible luciferase activity, further confirming the importance of this glycan for in vitro fusion activity of RSV F. Between the parental protein and the other glycomutants, no remarkable differences could be observed. 

### 3.3. Effect of Loss of RSV F N-Glycosylation Sites on Antibody Responses

**Figure 6:**
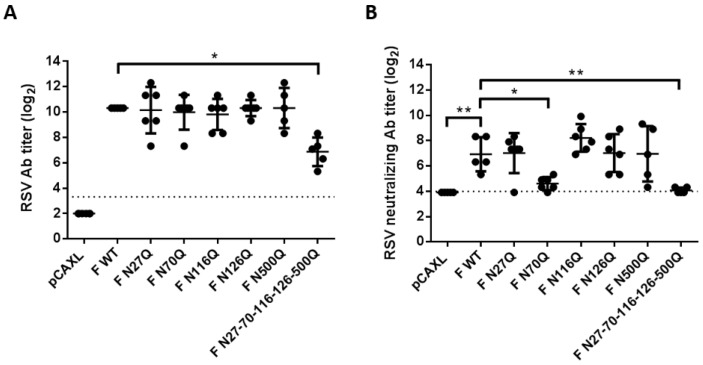
Antibody responses after DNA immunization of BALB/c mice. Two subsequent immunizations of the indicated plasmids were intramuscularly administered to BALB/c mice. Serum was collected 2 weeks after the second immunization. (**A**) HEp-2 monolayers were infected with RSV A2-K-line19F for 24 h and methanol fixed afterwards. Serum antibody titres were determined by titration of 2-fold serial dilutions of heat-inactivated serum. Binding of the antibodies was detected by HRP-conjugated goat anti-mouse IgG. Endpoint titres were determined by light microscopic analysis. (**B**) PRNT were performed to determine neutralizing antibody responses after boost immunization. Serial two-fold dilutions of heat-inactivated serum were incubated with RSV A2-K-line19F for 1 h at 37 °C prior to inoculation of HEp-2 monolayers. Plaques were visualized by immunostaining with palivizumab and HRP-conjugated secondary antibodies. The 50% endpoint titres were determined by manual plaque counting. The dotted line represents the detection limit. * *p* < 0.05; ** *p* < 0.01; n = 5–6 animals/group (Student’s unpaired two-tailed *t* test).

For multiple viruses, removal of N-glycans of viral proteins was shown to enhance humoral immunity after DNA immunization [25,26,27,28]. Since the RSV F protein is known as the most important target of neutralizing antibodies responses and therefore the major focus of vaccine development, the impact of the loss of N-glycosylation sites on immunogenicity was characterized in more detail. Therefore 6–7 weeks old female BALB/c mice were intramuscularly immunized with plasmid DNA encoding glycomutant RSV F proteins. Two consecutive immunizations were performed with an interval of three weeks. Serum was analysed to follow up the antibody titres as well as the neutralizing antibody titres (Figure 6A,B). In general, all F constructs induced serum IgG antibodies that specifically reacted with fixed RSV-infected HEp-2 cells and antibody titres increased after a second boost immunization. Significantly lower antibody titres compared to F WT were observed after immunization with plasmid DNA encoding pentamutant RSV F proteins (F N27-70-116-126-500Q). For the other glycomutants, no remarkable differences in mean antibody titres were observed. Interestingly, in plaque reduction neutralization tests (PRNT) the neutralizing antibody titres in serum from mice that had been immunized with F N27-70-116-126-500Q DNA or F N70Q DNA were significantly lower than those in the other F groups. 

### 3.4. Immunization with F N116Q DNA Results in Enhanced Neutralizing Antibody Responses and Protection upon Challenge

**Figure 7:**
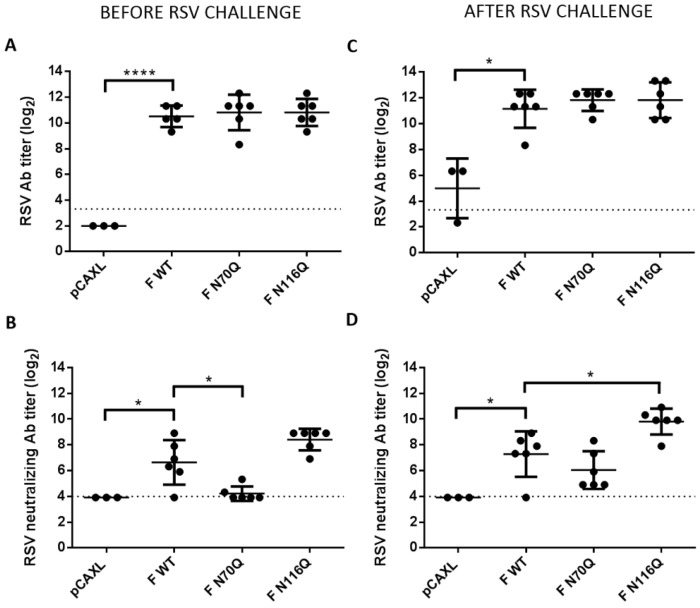
Antibody responses before and after challenge of immunized mice. Antibody (**A**) and neutralizing antibody responses (**B**) after immunization and before infection. Antibody (**C**) and neutralizing antibody responses (**D**) after immunization and after infection. To determine serum antibody titres RSV A2-K-line19F infected HEp-2 monolayers were titrated with 2-fold serial dilutions of heat-inactivated serum after methanol fixation. Binding of the antibodies was detected by HRP-conjugated goat anti-mouse IgG and DAB. Endpoint titres were determined by light microscopic analysis. (**B**) Plaque reduction neutralization test (PRNT) was performed by incubation of serial two-fold dilutions of heat-inactivated serum with RSV A2-K-line19F for 1 h at 37 °C prior to inoculation of HEp-2 monolayers. Plaques were visualized by immunostaining with palivizumab and HRP-conjugated secondary antibodies. The 50% endpoint titres were determined by manual plaque counting. * *p* < 0.05, **** *p* < 0.0001; n = 3–6 animals/group (Student’s unpaired two-tailed *t* test).

Finally, the efficacy of DNA immunization to clear viral infection and provide protection was evaluated by determination of the lung viral load after RSV challenge. Based on the previous findings, we selected a subset of the RSV F constructs for the challenge experiment, that is, mice that had been immunized with plasmids which elicited somewhat higher (F N116Q DNA) or significantly lower (F N70Q DNA) neutralizing titres compared to F WT, next to an empty vector control group (pCAXL) for challenge with RSV A2-K-line19F by intranasal inoculation five weeks after boost immunization. Antibody titres were determined before and after RSV challenge. Before the challenge, no distinction in anti-RSV serum responses could be observed between the different RSV F groups (Figure 7A). In contrast, as observed in the previous experiment, the neutralizing antibody titres elicited by F N70Q DNA were hardly above the detection limit before challenge (Figure 7B). Higher neutralizing capacity was observed for antibodies elicited after F WT and F N116Q DNA immunization with high and low varying levels, respectively. Five days post-challenge neutralizing antibody titres increased in mice that had been immunized with F N70Q and F N116Q DNA (*p* < 0.05, Mann-Whitney test) (Figure 7C,D; Figure S3). Significantly higher neutralizing titres were observed post-challenge for F N116Q DNA immunized compared to F WT DNA immunized mice. F N70Q neutralizing titres also increased after challenge, indicating some extent of priming after immunization but remained generally lower. 

**Figure 8:**
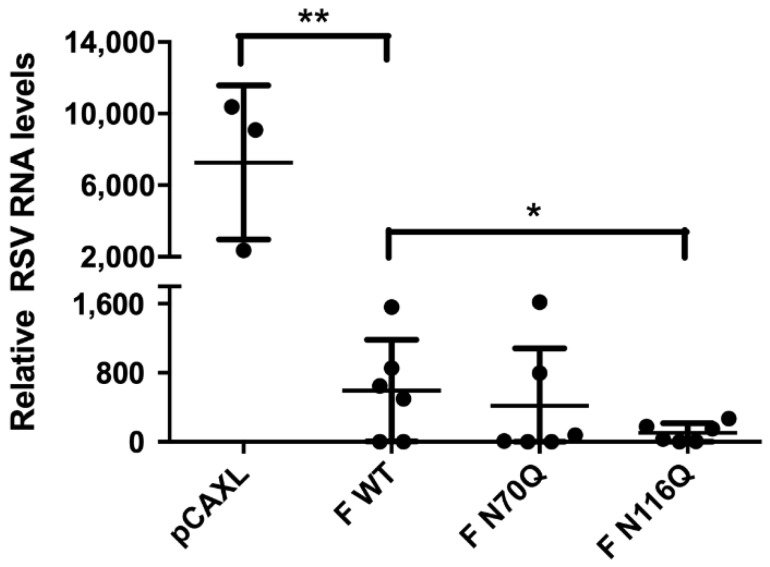
Lung viral RNA levels after RSV challenge. Five days post infection of the immunized mice with 1 × 10^6^ PFU of RSV A2-K-line19F, the left lungs were collected and homogenized. To determine relative RNA levels in the infected lungs RT-qPCR was performed. * *p* < 0.05, ** *p* < 0.01; n = 3–6 animals/group (Student’s unpaired one-tailed *t* test).

To which extent DNA immunization with vectors encoding the RSV F protein is able to clear lung viral infection in mice and how this is influenced by N-glycosylation of the protein, viral RNA loads were quantified by RT-qPCR. Except for the negative control, viral titres in lung homogenates could not be determined by a traditional viral plaque assay due to the presence of neutralizing antibodies in the lungs of the mice. Overall, RSV RNA levels were remarkably reduced compared with mice immunized with an empty plasmid (Figure 8). The lowest levels were observed for F N116Q, corresponding with the high serum neutralizing antibody titres in this group. Interestingly, despite the low neutralizing titres of F N70Q, similar levels of RSV RNA compared to F WT were observed here. 

**Figure 9:**
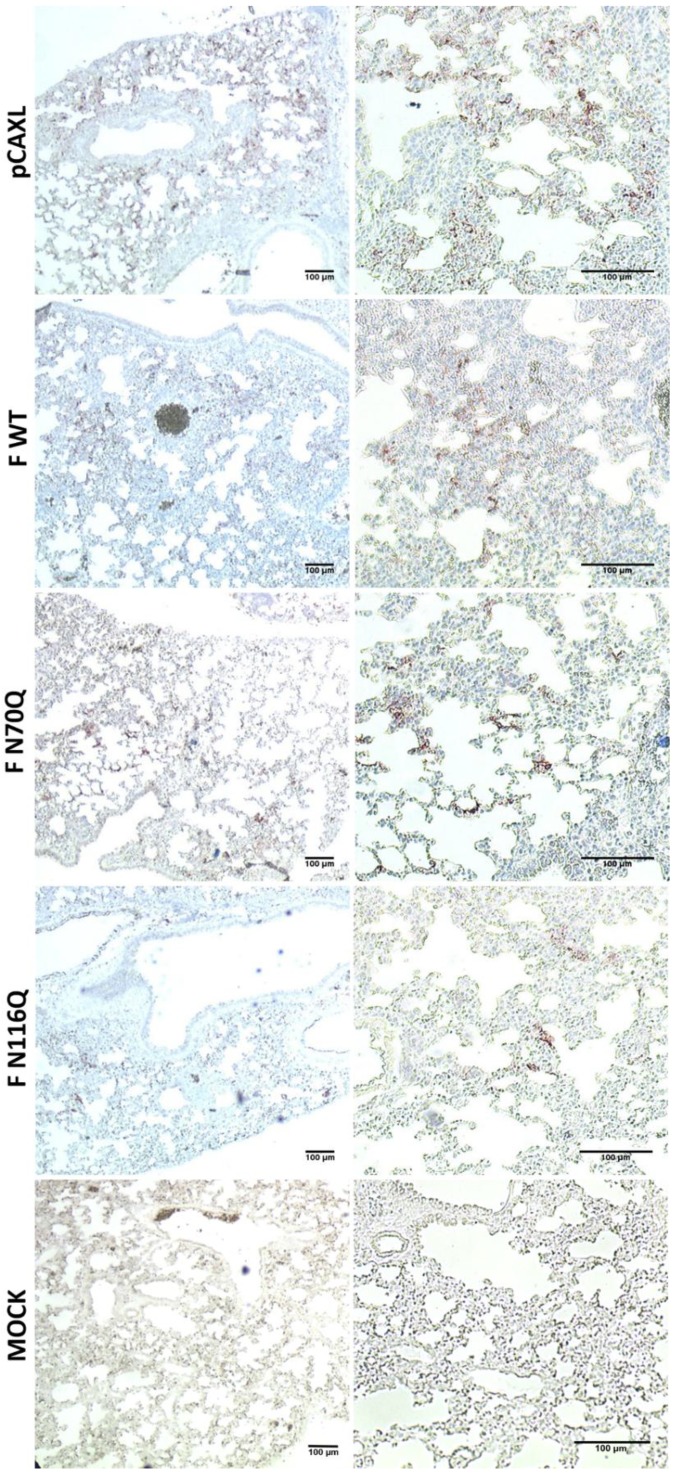
Immunohistochemical staining of PF-fixed, paraffin-embedded lung tissue for RSV antigen. Five days post infection, the right lungs were collected and fixed with 4% formaldehyde (pH 7.4) overnight and further incubated in 60% isopropanol. Lung tissues were paraffin-embedded and stained with goat anti-RSV IgG. Red staining indicates the presence of RSV antigen. Representative images are shown of the indicated groups of immunized mice (**left** column: 5×, **right** column: 10×).

Immunohistochemical staining of lung tissue for RSV antigens showed a good correlation between the viral RNA levels in the infected lungs and the presence of RSV antigens in the fixed lungs (Figure 9).

## 4. Discussion

Extensive research already showed the importance of glycosylation in the biology and function of a large number of viral proteins [18,20,21,22,44]. Due to its ability to promote survival and virulence by modulation of virus infectivity and pathogenicity and by interference with antiviral immune responses, glycosylation has become an area of growing interest. Since the N-glycosylation sites of RSV F are highly conserved, a determinant role in the structure or functionality of the protein is very likely as well as in the antigenicity of the protein. The sites are well-identified and certain characteristics were described [11,15,45,46] but gaps remain in the knowledge about hRSV F glycosylation, in particular in its relation to RSV F immunogenicity. Since the protein is the major antigenic target for RSV vaccine development this might be of importance. In this study, different N-glycosylation mutants of the RSV F protein were made and the effect on different characteristics was studied (Table 1). 

In order to characterize the RSV F glycosylation at protein level, the N codon was substituted by the Q codon at the 5 potential N-glycosylation sequons of the RSV F protein of strain line19 that are conserved among RSV isolates [47]. The presence of N-glycans at positions N27 and N500 was demonstrated by Western blot analysis since a reduction in molecular weight was observed. For F N70Q the reduction was less clear. No differences in molecular weight were observed for mutants F N116Q and F N126Q, likely because in the protein that was detected, p27 is lacking [15]. Previous studies suggested no role of glycosylation in RSV F cell surface transport [11,15]. It was presumed that cleavage of F0 might be required for surface expression and that glycosylation only facilitates cleavage through generation of a more stabilized conformation of F0 [11]. In our study, all glycomutants were efficiently cleaved from F0 precursors to disulphide-linked F1-F2 mature proteins and expressed at the surface, however reduced levels were observed for F N70Q and especially for F N27-70-500Q and F N27-70-116-126-500Q, probably due to lower expression of the proteins. As N-glycosylation is a prerequisite for correct folding, detection of low expression levels of the latter could be explained by the misfolding of these proteins after removal of multiple N-glycosylation sites. Remarkably, for F N500Q no significant reduction in surface expression was observed but total expression levels were clearly reduced.

The role of glycosylation in RSV F fusion activity was already shown by Zimmer et al. [15]. In this study, beside scoring syncytia formation after transfection, a more sensitive assay was used based on the reconstitution of Renilla luciferase after fusion of RSV F transfected cells. Both assays confirmed a determinant role of the N-glycan at position N500 in RSV F fusion while the other N-glycosylation sites seemed to be dispensable for fusion activity [15,23]. It was hypothesized that the position of N500 within the heptad repeat region could explain the importance for efficient fusion [15] or alternatively, N500 could be involved in the conformational reorganization from prefusion to postfusion conformation that takes place during the fusion process. 

Further characterization of the RSV F glycomutants was performed by studying the influence of glycosylation in the recognition of a panel of mAbs directed against well-defined epitopes of the RSV F protein [36,37,38,39,40]. Deletion of N-glycans can result in improved recognition by making the epitopes more accessible or alternatively, influence recognition negatively by modifying the protein conformation. After normalization towards the surface expression levels determined by a RSV-specific polyclonal antiserum, only for glycomutants F N70Q and F N500Q, reductions in mAb recognition were observed. This suggests that the neutralizing epitopes of the mAbs tested do not rely on glycosylation of positions N27Q, N116Q and N126Q. 

How glycosylation can affect the immunogenicity of a viral protein is well-studied for numerous viruses [25,26,27,28,29,48,49]. To our knowledge, this is the first report studying the impact of loss of individual N-glycosylation sites of the hRSV F protein on antibody responses by immunization of BALB/c mice with plasmids encoding RSV F glycomutants. Previous research already showed the potential of RSV F DNA immunization in mice to induce in vitro neutralizing antibody responses [50,51,52,53] which was also obtained in our approach using codon-optimized RSV F sequences. Based on the antibody titres after two subsequent immunizations, a role of N-glycosylation at position N70 in the induction of neutralizing antibody responses could be suggested. Interestingly, N70 is positioned near the apex of RSV F in close proximity of antigenic site Ø, which is a major target of highly potent mAbs in human sera [7,54]. The absence of the glycan at position N70 might possibly disturb this site, explaining the low neutralizing titres induced after immunization with F N70Q DNA. Furthermore, glycans themselves can be part of an antigenic site, as demonstrated for specific broadly neutralizing HIV mAbs [55]. Impaired total and surface expression of F N70Q could also explain these observations. However, total antibody responses of F N70Q immunized mice are comparable with F WT DNA immunization. Immunization with F N27-70-116-126-500Q DNA induced low antibody titres with almost no neutralizing activity which might be due to lower expression levels and malfolding of the protein.

RT-qPCR of lung viral RNA after RSV challenge showed partial clearance of pulmonary RSV in mice immunized with plasmids encoding the RSV F protein compared with lung RNA levels after immunization with an empty vector due to priming of higher neutralizing antibody responses compared to titres induced by natural infection. Removal of N-glycans can result in unmasking of neutralizing epitopes which was demonstrated for the highly glycosylated HIV Env protein and the HCV envelope protein [56,57]. Unexpectedly, higher titres and more efficient clearance were obtained by F N116Q DNA immunization, a N-glycan located in p27. For RSV, the effect of the removal of the N116 glycan would thus not be expected to unmask a neutralizing epitope, since the p27 is generally accepted to be absent on the mature RSV F protein because of two cleavages by furin during F protein maturation. Furthermore, blocking p27 cleavage using a furin inhibitor abolishes expression of the full length F protein (Figure S1). Partial cleavage of the mature RSV F protein, followed by a second cleavage event of RSV F during viral entry has however also been suggested and as such the p27 could be exposed to the immune system [58]. Clearly, further research will be needed to confirm this. Alternatively, since N-glycosylation is known as an important determinant for proper folding and processing of glycoproteins [18,59], deglycosylation can result in conformational changes and affect the accessibility of the neutralizing epitopes on the protein. Krarup et al. suggested a potential role for p27 in ensuring proper trimer formation [60]. According to this hypothesis, deglycosylation of this peptide may influence trimerization whereby conformational changes could affect exposure of neutralizing epitopes.

Nearly the same level of protection was acquired for the F WT and F N70Q DNA immunized groups, despite the low neutralizing titres of the latter group. This can be due to the requirement of a minimum of neutralizing activity, elicited by F N70Q, to provide this level of protection. On the other hand, this might be a consequence of other antibody mechanisms or immune responses that were induced after immunization and potentially upregulated after deglycosylation. High antibody titres were elicited after F N70Q DNA immunization suggesting a potential role of antibody-effector mechanisms that require Fc receptors such as antibody-dependent cell-mediated cytotoxicity (ADCC). Recently, RSV G-specific antibodies were identified in human serum whose in vitro neutralization is mediated by this mechanism [61]. In addition, protection provided by antibodies induced after vaccination of mice with the extracellular domain of the RSV SH protein (SHe) was also shown to depend on Fcγ receptors [62]. Here we analysed only neutralizing antibody responses but viral N-glycosylation can also affect the efficacy of cytotoxic T-cell (CTL) responses [63,64]. Proteolysis to form peptides or glycopeptides and recognition of T cell receptors by MHC molecules can be affected by glycans positioned near proteolytic sites [65]. Additionally, previous immunization experiments showed upregulation of Th1 cell responses after immunization of mice with a full-length RSV F DNA vaccine [51,52].

Extensive research to develop a safe and immunogenic RSV vaccine has been carried out since many years. Plasmid DNA and gene-based vaccines have the advantage to induce humoral and cellular immune responses, which are both important to provide protection against severe RSV infections [66,67]. DNA immunization is a promising vaccine approach with high stability levels and is easy for large-scale production. Nevertheless, poor immunogenicity remains a major struggle in their development as well as the safety and regulatory issues [68]. For RSV, few gene-based vaccines are currently in preclinical development and a couple candidates are in clinical phase I and II. Therefore, it might be more promising to evaluate our findings regarding the role of glycosylation in RSV F immunogenicity in gene-based vectors. Also in the context of other vaccine approaches such as RSV F based subunit vaccines and LAVs, our findings could be potentially an added value but requires more extensive research with soluble RSV F proteins and RSV infectious clones respectively.

In conclusion, our study showed enhanced antibody responses, especially after challenge, when BALB/c mice were immunized with a F DNA construct that lacks the N-glycan sequon for N116 located in p27. This is surprising because this glycan is not present on the mature RSV F protein, which lacks p27 and deglycosylation may impact the conformation of the protein before cleavage. Further research is needed to uncover the mechanism through which deletion of N116 results in enhanced exposure or potentially unmasking of epitopes and how this mutation could be implemented in potential vaccine candidates.

## Figures and Tables

**Table 1:** Overview of RSV F glycomutants. =, comparable to F WT; +, higher then F WT; −, lower then F WT.

	Surface Expression	Total Expression	% Transfected Cells	Fusion Capacity	Total Ab Response	Neutralizing Ab Response
**F N27Q**	=	=	=	=	=	=
**F N70Q**	–	–	=	= ^1^	=	−
**F N116Q**	=	=	=	= ^1^	=	+
**F N126Q**	=	−	=	=	=	=
**F N500Q**	–	−	–	−	=	=

^1^ Improved fusion activity was observed with the fusion assay based on syncytia formation.

## Supplementary Materials

The following are available online at http://www.mdpi.com/1999-4915/10/8/426/s1, Figure S1: Effect of furin inhibitor on RSV F expression and cleavage. Figure S2: Western blot analysis of the glycomutant F protein expression in a human cell line (HEK-T). Figure S3: Direct comparison of the total (left graph) and neutralizing (right graph) antibody titers before (B) and after (A) challenge of immunized mice.
