# Supplementary material for: Removal of the N-Glycosylation Sequon at Position N116 Located in p27 of the Respiratory Syncytial Virus Fusion Protein Elicits Enhanced Antibody Responses after DNA Immunization
Source: Viruses. 2018 Aug 14;10(8):426. doi: 10.3390/v10080426 (PMC6115940; doi:10.3390/v10080426)
Supplement: Supplementary file 1 [file viruses-10-00426-s001.docx]

Supplementary Data


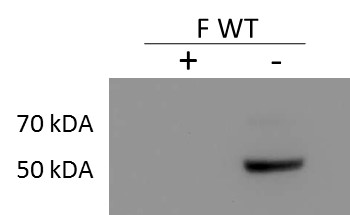


**Figure S1. Effect of furin inhibitor on RSV F expression and cleavage.** Cells were treated with the furin inhibitor decanoyl-R-V-K-R-cmk (100 mM, Tocris Bioscience) before and during transfection with F plasmid DNA. After cell lysis, the samples were loaded on SDS-PAGE under reducing conditions, followed by Western blot analysis using Palivizumab mAb. However, no detectable levels of uncleaved F proteins could be observed after treatment with the inhibitor. A non-treated sample showed a clear band at 50 kDA, representative for the F1 unit of the cleaved and mature F protein. The experiment was repeated once and resulted in the same result. Comparable results were observed in a previous report, were Krarup et al., observed no detectable levels of the full-length F protein after mutation of both cleavage sites (Krarup, et al. 2015).


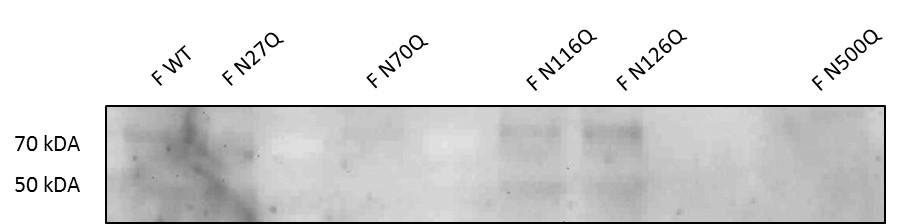


**Figure S2. Western blot analysis of the glycomutant F protein expression in a human cell line (HEK-T).** HEK-T cells were transfected with the WT and the different glycomutant F constructs. After 24 h incubation, the cells were lysed and the proteins were denatured. Finally, the samples were loaded on an SDS-PAGE gel and analyzed by Western blotting. The molecular weights observed for F WT, mutants F N27Q, F N116Q and F N126Q were similar to those of the respective F proteins expressed in BSR T7/5 cells. The bands of mutant proteins F N70Q and F N500Q could not be detected, probably due to lower expression levels of these proteins which was also observed after transfection in BSR T7/5 cells.

|  |  |
| --- | --- |
| (**A**) | (**B**) |

**Figure S3.** Direct comparison of the total (left graph) and neutralizing (right graph) antibody titers before (B) and after (A) challenge of immunized mice. For the total antibody titers, no significant differences where observed for any of the groups. In case of the neutralizing titers, a significant increase post-challenge was observed for groups N70Q and N116Q. *, P < 0.05 (Mann-Whitney test).

References

Krarup, A., et al. 2015 A highly stable prefusion RSV F vaccine derived from structural analysis of the fusion mechanism. Nat Commun 6:8143
